# Characteristics of Food Printing Inks and Their Impact on Selected Product Properties

**DOI:** 10.3390/foods14030393

**Published:** 2025-01-25

**Authors:** Zuzanna Domżalska, Ewa Jakubczyk

**Affiliations:** Department of Food Engineering and Process Management, Institute of Food Sciences, Warsaw University of Life Sciences, 02-776 Warsaw, Poland; s208829@sggw.edu.pl

**Keywords:** food inks, 3D printing, digital model, texture, rheology

## Abstract

Three-dimensional printing, or additive manufacturing, produces three-dimensional objects using a digital model. Its utilisation has been observed across various industries, including the food industry. Technology offers a wide range of possibilities in this field, including creating innovative products with unique compositions, shapes, and textures. A significant challenge in 3D printing is the development of the optimal ink composition. These inks must possess the appropriate rheology and texture for printing and meet nutritional and sensory requirements. The rheological properties of inks play a pivotal role in the printing process, influencing the formation of stable structures. This article comprehensively characterises food inks, distinguishing two primary categories and their respective subgroups. The first category encompasses non-natively extrudable inks, including plant-based inks derived from fruits and vegetables and meat-based inks. The second category comprises natively extrudable inks, encompassing dairy-based, hydrogel-based, and confectionary-based inks. The product properties of rheology, texture, fidelity, and printing stability are then discussed. Finally, the innovative use of food inks is shown.

## 1. Introduction

There has been a notable surge in research activities related to 3D food printing in recent years. This trend is expected to persist over the forthcoming 5–10-year period. It is anticipated that there will be a greater integration of professional 3D printers, innovative food ingredients, advanced design processes, and printing itself. The global population is projected to reach 9.7 billion by 2050 [1]. The combined effects of population growth, climate change, water shortages, and the problem of food waste present a significant threat to global food security. Given these challenges, 3D food printing technology could prove crucial in ensuring a food supply for a growing population [2].

Three-dimensional food printing technology is a significant innovation that has the potential to revolutionise the culinary market. It offers a unique opportunity to create complex and aesthetic dishes that go beyond the limitations of traditional cooking methods. Three-dimensional printers may become the future technology, allowing us to “print and eat” [2]. In 2006, the Fab@Home 1 3D printer was developed as an open-source printer that can create moulds using various printing materials. It was initially used to make chocolate, Nutella, and icing shapes. Since then, researchers at the University of Exeter, TNO, Stratasys, and elsewhere have developed 3D printing using other food materials [3].

Three-dimensional printing in food production offers the opportunity to create personalised products for consumers, which can help solve food availability issues, increase supply-chain efficiency, and enable products to take on a variety of complex shapes [4].

The 3D food printing process can be broken down into different steps. The desired object is initially digitally created using 3D scanning or computer-aided design (CAD). The raw materials, including gels, pastes, or powders, are carefully selected to ensure that they are compatible with the required printing conditions. The printer then draws the prepared material into syringes or cartridges. A series of layers of food are created. Once the food has been printed, it can be further processed to improve its texture, shape, or taste. This can include heating, cooling, or drying [5].

One of the most important advantages of 3D printing is the ability to personalise food according to the individual requirements and preferences of the consumer, allowing the production of meals with precisely tailored nutritional properties. This possibility significantly benefits people who limit or eliminate certain ingredients from their diet. The technique is also cost-effective and efficient, easy to transport, contains new ingredients, and has an extended shelf life [6].

Available research on three-dimensional printing in the domain of food has encompassed the following subjects: rheological characterisation and printability analysis [7], the impact of hydrocolloids on 3D printing processes [8], the modification of texture in foods through 3D printing [9], the development of edible 3D printing inks for patients with dysphagia [10]. Review articles have concentrated on advances in research into 3D printing technology in functional foods [11], printing the future food [5], trends and roles in achieving sustainability goals in the food industry [12], and 3D printing materials [13] and methods [14,15]. However, limited research in the literature has examined the characteristics of food inks and their impact on specific product attributes.

This article aims to analyse the composition of food inks and printing parameters that affect printed products’ quality, precision, and stability. The focus is on the influence of ink’s composition on its rheological properties, texture stability, and print fidelity. Selecting appropriate printing parameters, such as the process temperature and printing speed, and using the proper nozzle diameter and height significantly impact the quality of the final 3D-printed product. Understanding how different material components and process parameters affect the final product’s properties is essential to improving current technologies and developing new and innovative solutions. The article also presents several creative applications for food inks.

## 2. Printing Inks in Food Production

Food-grade material, called “food ink”, is a key component of 3D food printing technology. The properties of these materials must allow them to flow through the nozzle and stabilise once deposited on the surface. Therefore, it is possible to control the viscosity and flavour of the food-grade materials used in food inks. Additional ingredients incorporated into the base products to improve their physical properties or provide nutritional benefits can be divided into four main categories: carbohydrates, proteins, fats, and food viscosity enhancers [12]. Food inks have been developed to personalise 3D-printed food. The food ink market offers many possibilities, including changing texture, taste, satiety, and appearance, and it contributes to changing eating habits. New technologies and consumer attention drive the development of food inks [16].

**Figure 1:**
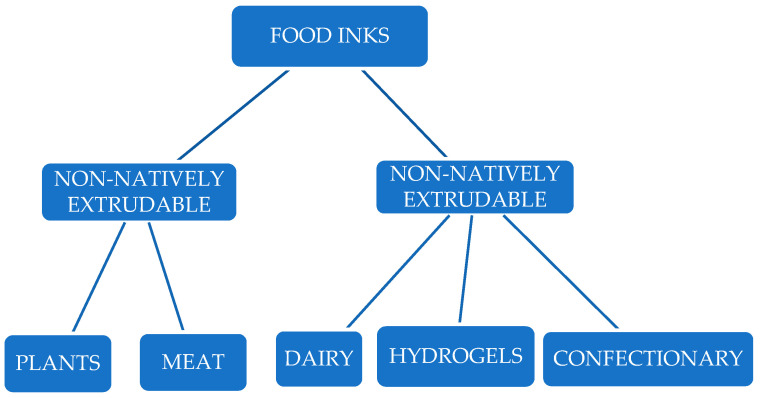
The categorisation of food inks according to Voon et al. [18].

Printing materials can be divided into three main categories: native printing materials (e.g., cheese), alternative materials (e.g., insects), and non-native ingredients (e.g., meat). Some printing materials can be easily extruded through nozzles without additional flow enhancers and exhibit a stable structure after printing. Unfortunately, changes in physical and rheological properties may be necessary when using non-native materials to achieve the required printability and smooth extrusion. Consideration may be given to incorporating new sources of beneficial ingredients, such as alternative ingredients (fibres and proteins), into other inks to produce specialised food products of a given shape [17]. Figure 1 shows one of the most commonly used classifications of food inks [18]. This classification includes materials that can be printed directly (natively extrudable inks) or those that require special preparation to be printed (non-natively extrudable inks).

Some inks are classified according to their primary ingredients, including protein-, fat-, and carbohydrate-based inks. Lille et al. [19] investigated the potential of 3D printing to create foods high in protein and fibre while reducing sugar and fat content. The ingredients used included the main nutrient groups (protein, fat, fibre, and carbohydrates). The materials used included faba bean and oat protein concentrate, milk powder, rye bran, winter-swelling starch, and cellulose nanolayers. This research is crucial for the use of 3D printing in the development of foods with essential nutrients.

Table 1 presents the composition of inks and parameters applied during 3D printing. The inks are based on plant, meat, dairy, and bakery ingredients. To ensure the stability of the inks during printing and storage, various types of stabilising and thickening substances are added to the inks, mainly hydrocolloids. Printing parameters such as the nozzle diameter, printing speed, and temperature of the process are also changed.

### 2.1. Non-Natively Extrudable

#### 2.1.1. Plant-Based Inks (Fruits and Vegetables)

Fruits and vegetables are a rich source of carbohydrates, vitamins, minerals, and other nutrients, meeting the nutritional needs of the entire population. They are characterised by nutritional, health, and economic value, making them essential to a balanced diet [37]. The direct suitability of fruits and vegetables for 3D printing is limited by their inherent flow and viscosity properties, which can be enhanced by adding appropriate additives such as starch, hydrocolloids, or hydrogels. In addition, high-value ingredients can be used, including functional ingredients and bioactive chemicals derived from plant extracts, bioactive peptides, fibres, algae, and phytochemicals from food waste. Probiotic strains are a new addition to the formulation of 3D inks. Fruit juices and beverages can be enriched with bioactive chemicals and probiotics, but designing them for 3D printing is a significant challenge. One study printed fruit and vegetable smoothies with added fish collagen to increase the viscosity of the paste. However, these beverages could be destabilised when lipophilic compounds and probiotics were added, which poses a significant obstacle to the broader application of 3D printing [38].

Yang et al. [35] successfully used 3D printing technology to create lemon juice gels with different starch contents of 10, 12.5, 15, 17.5, and 20%. The gelling agent used in the study was starch, which has beneficial water-retention, ageing-resistance, and transparency properties. Derossi et al. [39] developed fruit-based snacks for children. They can provide children aged 3–10 years with 5–10% of their energy, iron, calcium, and vitamin D requirements. Severini et al. [40] assessed the feasibility of building edible objects from various fresh fruits and vegetables, including carrots, broccoli leaves, kiwis, pears, and avocados. They built pyramid-shaped edible objects with an appearance that they liked better than a “smoothie” without printing. Three-dimensional printing did not significantly change the sensory characteristics of the samples or the antioxidant capacity and total phenolic content.

Pant et al. [27] printed food inks from garden peas (80% water content), pak choi (96%), and carrots (90%), which differed in the addition of hydrocolloids such as xanthan gum (percentage content ranging from 0 to 1%), locust bean gum (0–2%), kappa carrageenan (0–0.7%), and water content. Consequently, it was concluded that the higher the percentage of starch and the higher the water content in the vegetable were, the lower the proportion of hydrocolloids that could be added to the inks was. For example, garden peas with a water content of 80%, i.e., the starchiest of all vegetables tested, could be printed without adding hydrocolloids acting as stabilisers and thickeners.

Zhou et al. [41] developed an ink mainly composed of *Aronia melanocarpa* fruit pulp (AM) mixed with methylcellulose (MC), hyaluronic acid (HA), and pea albumin (PA) in precise proportions. The AM:MC:PA:HA combination ink at a ratio of 100:14:1:1 exhibited high printing precision and excellent physical and rheological properties and achieved ideal 3D printing suitability compared to other proportions and samples that had been previously printed.

The main factor hindering the widespread use of fruits and vegetables in 3D printing is their intrinsic material properties. The high water content of these products reduces the viscosity of edible inks. Two methods can be used to reduce the moisture content of these food materials. The first involves adding significant thickeners, including proteins, starches, and hydrocolloids. The second involves using various dehydration techniques to reduce the moisture content [13]. In addition, 3D printing technology can potentially improve the visual appeal of fruit and vegetable products, which consumers often perceive as unattractive. This can be achieved by printing mixtures of different ingredients and modifying the taste, aroma, or colour by adding small amounts of more acceptable ingredients. For example, children often do not like broccoli or kiwi despite their antioxidant content. The use of 3D printing can make them more attractive. In addition, this technology is effective in producing soft food products for consumers with dysphagia [42]. Dysphagia, or difficulty swallowing, is a condition in which patients experience difficulty or discomfort when chewing and eating food. In response to these problems, specially modified food textures, such as thickened liquids, smooth fruit and vegetable purées, and soft, moist, lumpy foods, are available to make eating easier. Choosing the right consistency of food is of great importance for people with dysphagia, as the wrong consistency of food can pose a risk of choking or asphyxiation [2]. The foods used in diets for consumers with dysphagia are in the form of pastes, purées, or viscous liquids, which can result in a visually unappealing presentation and a consequent reduction in appetite. Three-dimensional printing has emerged as a promising method for developing diets that are not only appetising but also safe, thereby facilitating the act of swallowing [43]. Severini et al. [40] demonstrated the feasibility of using fruit-based inks to provide 5–10% of the energy, iron, calcium, and vitamin D requirements for children aged 3–10 years. Three-dimensional printing technology is considerably promising for incorporating easily degradable bioactive compounds and other functional ingredients into food products. Consequently, it has considerable potential to enhance the consumption of fruit and vegetables, recognised as functional foods promoting human health [38]. This technology can also increase waste-free uses of fruit and vegetables and reduce food waste. Additionally, 3D printing (mainly extrusion methods) could use wastes such as mango and orange peels, apple and pineapple seeds, durian husks, and the stems of spinach and kale stalks. Wastes from plant materials contain many bioactive compounds that can be extracted and used to produce new enriched foods [44].

#### 2.1.2. Meat-Based Inks

Meat and meat products are nutritionally rich foods with deep cultural, social, and culinary traditions. They provide high-quality protein and are a rich source of many minerals and trace elements, including heme, iron, zinc, and selenium, as well as vitamins, especially B vitamins. They are also a source of long-chain fatty acids, including eicosapentaenoic acid (EPA) and docosahexaenoic acid (DHA), as well as bioactive compounds such as choline, carnitine, carnosine, and anserine [45]. However, the excessive consumption of meat products is associated with adverse health effects, mainly attributed to the presence of cholesterol and saturated fatty acids, which are associated with an increased risk of cardiovascular disease [46]. Conversely, substituting red meat for alternative protein sources can reduce the risk of coronary heart disease. The consumption of poultry in conjunction with a diet rich in vegetables has been linked to a reduced likelihood of overweight, obesity, cardiovascular disease, and type 2 diabetes. Consequently, a balanced diet constitutes a pivotal element of a healthy lifestyle [47].

Meat materials used in 3D printing can be understood as a multiphase colloidal system, where water is the continuous phase. At the same time, proteins, carbohydrates, and inorganic salts constitute the dispersed phase. The above system is distinguished by complex rheological properties, which make it unsuitable for printing procedures and pose a serious challenge to the development of 3D printing technology [48]. However, due to their low viscosity, these products can only be directly used in 3D printing technology with additional modifications. Various additives, including enzymes, are used to improve the properties of these materials and facilitate protein modification in some cases. For example, transglutaminase added in the meat printing process allows the assembly of proteins into self-supporting structures, thus increasing the stability and printability of meat pastes. Another additive used in 3D meat printing is gluconolactone (GDL), which acts as an acidifying agent [13].

The following section presents selected examples illustrating the use of meat in 3D printing. Liu et al. [49] developed a printer that uses a peristaltic pump to drive the material extrusion process. The use of a control panel allows independent control of the three axes. Compared to commercially available food printers, which are limited to using food materials like powder or granules to create pastes, the newly developed printer allows fibrous meat to be printed material. Studies that have been found to be effective include those by Hertafeld et al. [50] on printed materials made from shrimp paste and chicken paste, Lipton et al. [51] on printed turkey purée with added transglutaminase, and Li et al. [52] who showed that the addition of gellan gum and water had a significant effect on the physicochemical and rheological properties of chicken meat batters. Adding gellan gum in the amounts of 0.2% and 0.4% contributed to maintaining the stability of the dish during cooking and improving the textural properties of the chicken coating while at the same time reducing the pork fat content.

The unique fibrous composition of meat presents a challenge in the 3D printing process. Pre-treatment or the addition of appropriate and suitable products is necessary to facilitate the extrusion of the ink from the nozzle and to ensure the required self-support and formability [48]. It is worth noting that, in its various forms, meat is the least researched category of food inks [18]. Many studies have focused on meats with a solid fibre structure and low viscosity, such as beef or chicken breast. Therefore, to successfully implement this technology in meat production, printing materials must be further developed, and the range of meats that can be used in 3D printing must be expanded [38].

### 2.2. Natively Extrudable

#### 2.2.1. Dairy-Based Inks

Dairy products are a rich source of nutrients, including protein, calcium, riboflavin, iodine, phosphorus, and vitamin B12. However, the nutritional profile of dairy products varies depending on the specific product [53].

The mechanical and rheological properties of a given material are of great importance for its three-dimensional printing capabilities and the precision of the resulting geometric shape. With an increase in the protein content in the protein matrix, the internal structure of a milk protein gel transforms from an entangled form to a temporary three-dimensional network. The 3D printing quality is better for materials with a gel structure than materials with an entangled structure [54].

Lee et al. [55] investigated the influence of milk powder concentration on the rheological properties and printability of obtained material. It was shown that printing three-dimensional structures with milk inks containing 70–75 wt.% milk powder was feasible without additional rheological modifiers. The three-dimensional structures were produced by cold extrusion using a 3D printer with direct ink writing at room temperature. Cold extrusion has been used to produce nutritious and visually appealing food. This printing method at room temperature can preserve many nutrients in food products.

Other dairy ingredients, including whey protein and milk protein concentrates, have been used as raw materials for producing edible inks [56]. The use of dairy products for printing was discussed by Liu et al. [57], who showed, among others, that a protein paste prepared from a combination of cow’s milk protein concentrate (MPC) and whey protein isolate (WPI) in a ratio of 5:2 could be successfully printed and showed a higher degree of compliance with the designed 3D model. In contrast, Du et al. [58] showed that adding different whey protein derivatives affected a gel system, including its water distribution, rheological properties, texture, and microstructure changes. The whey protein content of less than 20% resulted in unsatisfactory printing performance for the gel system. In contrast, the gel with 20% whey protein added showed better printing performance.

The dairy category encompasses a wide range of commodities and ingredients with various compositions, functionalities, and structures, making dairy a promising source of raw materials for food printing. The potential for printing dairy products (e.g., cheese, yoghurt, butter) and ingredients (e.g., protein isolates, edible lactose, milk minerals) has not yet been thoroughly explored, despite their significant potential in food preparation applications [59].

#### 2.2.2. Hydrogels-Based Inks

Hydrogel products are polymeric materials that, due to their hydrophilic structure, can retain significant amounts of water in three-dimensional networks, which allows them to absorb and retain moisture [60]. Viscosity enhancers for 3D-printed food include many polysaccharides, such as guar gum, gum arabic, and carboxymethylcellulose [12].

In the food industry, hydrogels are primarily used as thickeners and stabilisers. For use in the food industry, hydrogels should be derived from plant biopolymers such as proteins and polysaccharides [61]. One of the first edible, printable hydrogels described in the literature combines two hydrocolloids (xanthan and gelatine) with added flavour concentrates, as reported by Cohen et al. [62]. This has enabled the creation of printable materials that simulate a variety of foods with a minimum number of ingredients. Research on hydrogels has increased significantly in recent years compared to the other food inks mentioned above.

Contemporary research on 3D-printed edible hydrogels (3DFP) typically has several goals, including developing theoretical models or simulations of the ink and printing process, developing printing technologies, optimising printing parameters, determining the effect of the type and/or concentration of different ingredients, and discovering new hydrogel inks [18].

A considerable amount of evidence has been gathered from numerous studies to support the claim that the addition of hydrogels improves 3D printing quality. Bulut and Candoğan showed that adding gelatine to a poultry-based snack significantly improved its printing properties. The introduction of gelatine at a concentration of 1.79% increased the extrudability of the product by modifying its viscoelastic properties. The product showed a higher overall quality, including sensory attributes, both before and after baking, with increased firmness, gumminess, and chewiness and reduced elasticity [63]. Rahman et al. [64] reported that the addition of konjac affected the network structure of agar, reducing the gel strength at break, which was confirmed by rheological tests. The study showed that increasing the concentration of konjac in the agar gel resulted in increased gel elasticity. Furthermore, it was observed that the addition of konjac had a beneficial effect on the 3D printing process of gel samples, as it facilitated the extrusion of a more significant amount of fluid, thus increasing the viscoelastic properties of the gel. Ishwarya et al. [61] demonstrated that pectin hydrogels have been shown to play an essential role in the food sector. The ability of the pectin hydrocolloid to expand under simulated gastrointestinal conditions makes it an effective satiety agent while maintaining its gel configuration. The viscoelastic properties and adaptable microstructure of the low-methoxylated pectin gel make it an optimal ink for three-dimensional printing. The complex relationship between its dimensions, structure, composition, and rheological properties makes it an intriguing food ingredient and a stand-alone product. The use of hydrogel-based inks in three-dimensional printing technology presents a promising avenue for the development of personalised food products. Nevertheless, challenges remain to be addressed, such as improving hydrogel inks’ printing performance and mechanical properties [65].

#### 2.2.3. Confectionary-Based Inks

Candy-based inks are composed primarily of chocolate-based ingredients. Chocolate is considered a printable material. Chocolate describes a thick suspension of non-fat particles, including sugar, milk powder, and cocoa solids, dispersed in a continuous phase of cocoa butter. In the molten state, chocolate exhibits shear thinning, thixotropy, and flow stress behaviour. These behaviours may vary depending on its formulation and particle size distribution [66]. The concept of printability is based on the ability of a given material to be extruded from a nozzle and to maintain its shape after being applied layer by layer. The parameters that must be considered when applying chocolate are the extrusion speed, the speed of the nozzle movement, and the distance from the printer table [67]. Chen and Mackley [68] were among the first to report on the observations of chocolate regarding the cold extrusion process and, consequently, its elasticity after extrusion. Isothermal cold extrusion below the material’s primary melting point increased the cocoa butter’s liquid fat content, which in turn led to a temporary increase in the elasticity of the chocolate. The extrusion process induced a phase change in the cocoa butter rather than merely heating the material.

Three-dimensional chocolate-printing technology is still developing and offering many advantages. These include the ability to create personalised shapes, customised diets, and medications. While extrusion is the preferred printing method, further research is needed to determine the feasibility of other techniques. A detailed study is required to demonstrate the optimal settings for each type of printing, as the settings need to be tailored to material properties. The most important thing is to ensure that the 3D-printed product undergoes a sufficient cooling process, as this significantly impacts the strength of the structure. Consumer opinions are promising, as there is considerable interest in the complex shapes that can be achieved with 3D printing [69].

Several studies have been published on chocolate-based inks. Karyappa and Hashimoto [70] demonstrated the feasibility of producing 3D models comprising chocolate syrups and pastes combined with cocoa powder at 10% to 25% by mass. In addition, chocolate-based models containing different chocolate inks (liquid filling and semi-solid shells) were also created. However, Mantihal et al. [57] investigated the effect of the shape and internal structure of 3D-printed chocolate models. They also evaluated the texture of the obtained products.

Another confectionery product subjected to analysis in several recent studies is cookie dough, typically composed of wheat flour, other standard ingredients (such as water, butter, and sugar), and additives such as powdered mealworm larvae [18]. Guénard-Lampron et al. [71] developed a cake recipe based on carrot purée. They investigated the effect of printing parameters, particularly the nozzle and filling speed, and post-processing parameters, including the time and temperature, on the resulting structure.

### 2.3. Printing Ingredients

Printable ingredients can also be classified in several alternative ways, including by dividing them into traditional and non-traditional food materials or categorising ingredients based on their protein, fat, and carbohydrate content [18].

Using protein as a 3D printing ink could revolutionise the food industry by enabling the production of nutritionally tailored foods for specific consumer groups, including the elderly, pregnant women, children, people with health conditions, and athletes. Protein is a rich source of essential and non-essential amino acids that offers many health benefits when used as a food printing material [72].

Casein and whey proteins are two types of proteins suitable for 3D printing. Casein and whey proteins differ in structure and function, and each family contains proteins with unique properties. Whey proteins, widely used in the food industry due to their excellent functional and nutritional properties, have a spherical structure. When heated to temperatures above 70 °C, they denature and aggregate, forming gels when their concentration is at least 6%. The gelation process of whey proteins depends on factors such as pH, temperature, heating time, and the presence of solvents. Gel formation occurs due to bonds between denatured proteins and non-covalent (e.g., hydrophobic) and covalent (e.g., disulphide) bridges [59].

The incorporation of enzymes into protein inks has a profound effect on their internal structure. Transglutaminase (TGase), a frequently used enzyme for forming protein gels, facilitates this process by catalysing the acyl transfer reaction between the γ-carboxamide group of glutamine and the amino group of lysine in other proteins. This reaction leads to intermolecular and intramolecular cross-links by generating ε-(γ-glutamyl)-lysine bonds [72].

Fats play a key role in 3D printing, with margarine, butter, and cooking oils being particularly popular [12]. The main vegetable inks are mainly derived from soybean oil and linseed oil. Soybean oil is the preferred choice due to its compatibility with the ink system and lower price. In addition, other vegetable oils are chosen for their characteristic chemical composition and unique properties [73].

Carbohydrate sources such as agar, flour, rice starch, potato starch, maltitol, isomaltose, and xylitol, as well as proteins such as edible insects, surimi, beans, peas, whey, and egg white, have specific properties that are conducive to the production of 3D food [12].

Inks derived from alternative ingredients can also be used for 3D printing. Such ingredients include algae, fungi, seaweed, lupine, and insects, providing a new source of protein and fibre. In addition, existing processes can convert other products into bioactive metabolites, enzymes, and flavour compounds [74].

## 3. Crucial Properties of Inks and Printed Foods

Food inks are a key element of 3D printing [12]. For an ink to be suitable for printing, it must meet several requirements. These include the ability to flow easily through the nozzle tip and maintain the product geometry through sufficient mechanical strength [4].

The properties of 3D-printed food differ from the materials used to produce it. In the case of food printing, the material from which the product is made must meet relevant standards and regulations regarding food safety. Therefore, it is essential to know the materials’ properties [75].

Ink with an inadequate elastic gel formulation, suboptimal thixotropic properties, and limited plasticity does not form a network structure. Such samples were evaluated by printing different geometric figures that showed spatial resolution deficiencies and a lack of shape uniformity (geometric irregularities) [4].

Particle size is essential, concerning the thickness of the printed layer, with thinner layers exhibiting better mechanical properties. Material characteristics related to mass and surface area, such as density and thermal properties, including thermal conductivity and diffusivity, heat resistance, and crystallisation temperature, significantly affect the structure of food obtained by the printing process. In addition, properties such as glass transition, viscosity, compressibility, dry behaviour, absorption, and permeability also play an essential role [75].

### 3.1. Rheological Properties and Texture

The rheological properties of raw food materials are of great importance in determining their flow during the printing process. This affects the viscosity and elasticity of the food during the extrusion process. It regulates the printed food product’s layer deposition and structural stability [5].

Rheology is essential in enhancing the extrusion of food products during printing and ensuring that they maintain their optimal form. In the context of 3D food printing, rheology plays a key role in determining the properties of the printing ink. The addition of konjac powder affects the cross-linked structure of agar, which causes a decrease in the gel strength. The introduction of konjac increases the elasticity of the agar gel network, increasing its concentration. The use of konjac in 3D printing gel samples has facilitated smooth extrusion by improving their viscoelastic properties [64]. In the context of 3D printing, the apparent viscosity of the inks used must be high enough to allow stacking with previous layers while at the same time being low enough to allow unimpeded extrusion through the tip and prevent clogging [76].

Flow stress and the storage modulus are key parameters that facilitate ink flow in the resting state and ensure the structural integrity of the printed material after printing. By adding milk, Lee et al. increased an ink’s viscosity from about 12 to 130 kPa·s due to milk powder’s addition. However, the viscosity decreased with an increasing shear rate, causing the ink to become pseudoplastic and shear-thinning. In addition, the plasticity rose from 0.08 to 1.5 kPa, indicating that the increase in milk powder concentration increased the colloidal network in the ink [26].

A raw material that is similarly characterised by high plasticity is hydrocolloids. Hydrocolloids are a class of food additives with a wide range of functions, mainly due to their ability to interact with and bind to water. The most commonly used hydrocolloids include starch, xanthan gum, beta-glucan, guar gum, locust bean gum, konjac, pectin, alginate, carrageenan, and inulin. They act as gelling, thickening, and stabilising agents. In 3D printing technology, hydrocolloids can be added to inks to adjust their rheological properties [77].

Adding hydrocolloids, which act as thickeners, plasticisers, and binders for food materials, is another aspect that improves the flow properties of 3D printing inks. Carrageenan, for example, is often incorporated into meat products to form a gel and retain water. Gelatine is commonly used as a hydrocolloid in 3D printing, serving as a modifier in selected food formulations. The rheological properties of chicken-based formulations showed that the addition of 1.79% gelatine significantly improved the printability of the product, resulting in a 110% filament flow rate, a 90% feed rate, and a 0.5 mm nozzle height [63]. Ensuring that the pastes used are non-Newtonian fluids is essential to guarantee proper 3D printing [76].

In the context of food inks, where particles are dispersed within a hydrocolloid matrix, the primary factors influencing rheological properties and printability are the type and content of the particles. The addition of vegetable powders (broccoli, spinach, carrot) at 10% and 30% concentrations to matrices with different hydration properties demonstrated that hydroxypropyl methylcellulose at a 30% powder content exhibited the most significant disparities in rheology and printability. In contrast to its higher hydration capacity, adding xanthan gum minimised these differences. The study also found that broccoli powder, due to its high hydration capacity, affected the rheological properties of the inks due to more particle swelling [78]. Ainis et al. [79] investigated the rheological properties of pea protein isolate (PPI) paste and soy protein isolates (SPIs), with the protein content of the isolates being 83% and 90%, respectively. Rheological parameters such as G’ and tanδ were found to be dependent on the protein type and concentration. The PPI exhibited higher tanδ values, which indicated reduced elasticity compared to the SPIs. The results showed that different proteins had a significant impact on the stability of the pastes. Furthermore, the relationship between elasticity and protein concentration was contingent on the specific type of protein. Tyupova and Harasym [44] presented that the texture of fruit inks could be assessed by their rheological properties, which mainly depended on the composition of, structure of, and relationship between polysaccharides, lipids, proteins, and water. Venkatachalam et al. [80] developed pea-based food inks for 3D printing, incorporating pea fibre, protein, and starch. The composition of the ink and the processing parameters had a significant impact on the cracking behaviour of the products. It was demonstrated that increasing the protein content and higher starch concentrations in 3D pastes increased fracture stress.

Texture is a physical property of sensory and structural components of food. Texture analysis is essential for understanding the structural integrity of three-dimensional-printed food products and assessing their related sensory properties and oral texture perception [81]. Most researchers use texture analysers in conjunction with texture profile analysis (TPA) to evaluate the texture of 3D-printed products. The parameters covered by this analysis include but are not limited to cohesion, viscosity, hardness, gumminess, chewiness, and elasticity. The most important indicators of structural integrity are cohesion, elasticity, and hardness, given their correlation with product strength. Cohesion, defined as the internal viscosity of a sample and its ability to deform before fracture, is a key factor in assessing the integrity of 3D-printed products [82].

Carranza et al. [83] demonstrated that incorporating red cabbage at varying proportions (0, 10, 20, and 30%) into soy protein isolate cakes influenced the textural characteristics of the products. Adding a higher red cabbage concentration resulted in the cookies’ hardness. As the filling speed increased, the energy required to prepare the food for swallowing also increased. In addition, the increase in filling speed caused more material settling and entanglement, which led to higher hardness, gumminess, and chewiness in the printed and hydrated samples. Kim et al. [84] noted that texture was additive-dependent for isolated pea protein and honey red ginseng. Zhao et al. [85] investigated the cohesion of tomato–starch paste samples containing wheat and potato starch and corn, tapioca, or mung bean starch samples. The more unusual ability of wheat and potato starch to form a dense, three-dimensional network when combined with tomato molecules resulted in higher cohesion in the first samples. Products printed from a paste based on wheat and potato starch showed the highest chewiness. In contrast, the lowest chewiness was observed in samples with added mung bean starch. Riantiningtyas et al. [86] emphasised the importance of collating rheology with texture by analysing yoghurt gels, whey protein isolate (WPI), and gelatine. The incorporation of 12% WPI led to a substantial reduction in the hardness of the gels before printing. In comparison, an augmentation in gelatine concentration from 7.5% to 12.5% resulted in a 100–115% escalation in hardness. The rheological data demonstrated that the more complex samples also exhibited a higher yield stress. Both components, the WPI and gelatine, influenced the sensory properties of the yoghurt gel. The WPI primarily enhanced the taste, aroma, and mouthfeel, while the gelatine had the most significant impact on the appearance and texture of the product.

In the field of 3D printing, achieving optimal results requires considering the properties of the materials. In particular, inks with higher hardness levels require higher extrusion pressures and motor speeds to prevent clogging and ensure smooth printing. Inks with higher hardness require precise control, while inks with lower hardness, such as those composed of softer materials, exhibit greater fluidity but may produce reduced structural stability. Improving ink cohesion promotes uniform flow and increases layer stability during printing, thereby promoting better precision and shape retention in the deposited material [87].

#### Modelling Rheological Properties

The modelling of rheological behaviour has played a crucial role in food engineering [88], especially in the design process of new products. The rheological properties of materials can vary from viscous liquids (with or without elasticity) to elastic solids [88,89]. These properties can be described by physically based models like the Maxwell, Kelvin–Voigt, and Burgers models [89]. Viscoelastic liquids are used in the Maxwell model, which contains a spring in series with a dashpot. The model indicates a sequence of elastic and viscous behaviour. The Kelvin–Voight model with a parallel connected spring and dashpot characterises the viscoelastic behaviour of solids. The Burgers model, as a combination of the Kelvin and Maxwell models, can applied to determine the transient properties of the viscoelastic behaviour of solids [90]. Jonkers et al. [91] applied a single Maxwell model to describe the material behaviour of a brittle 3D-printed mixture of starch, maltodextrin, and palm oil powder. Vancauwenberghe et al. [92] applied Gibson and Ashby’s analytical model and finite element model (the transient structural model of ANSYS) to predict the textural properties and porosity of 3D-printed pectin-based food simulants. The accuracy of model prediction depended on the accuracy of the printed structure. A validated finite element model enabled the design of a more complex structure. The finite element method can be used to simulate the behaviours of deformable materials. This method can be a helpful tool in evaluating quality and safety losses during the production and storage of food [89]. Partial least squares and back-propagation artificial neural network (BP-ANN) models based on low-field nuclear magnetic resonance data were used to predict the rheological properties of 3D-printed pineapple gels with different additions of maize starch and xanthan gum [93].

Many models can be used to describe the flow behaviour of Newtonian and non-Newtonian fluids. The relationship between the shear rate and shear stress can be used to characterise a shear-thinning flow without yield stress [94]. The power law model is frequently used to describe the rheological properties of non-Newtonian fluids. This model was applied to characterise the flow behaviour of complex formulations with ground dried orange peel, amaranth leaves, guava, whole milk, sugar cocoa powder, cricket flour, pregelatinized waxy corn starch, and water. The *K* parameter, related to consistency, varied from 190 to 476 Pas depending on the composition of inks. The *n* parameter of a model describing the pseudoplasticity for this ink was about 0.22. The viscosity and resistance to the flow of the samples increased with an increasing concertation of starch [95]. Low values in model parameters are recommended to obtain a steady flow of material during printing [78]. Shear-thinning behaviour was also observed in ink based on k-carrageenan, xanthan gum, and potato starch. The parameters of the power law model indicated that the consistency index K decreased with an increase in temperature from 35 to 40 °C. The addition of xanthan gum and potato starch reduced the n parameter’s value, indicating that a significantly shear-thinning ink was obtained [96]. This rheological behaviour of inks based on soy and pea proteins was also described using the power law model. Ink with soy protein was more elastic, which led to better stability against collapse during printing [79]. The Herschel–Bulkley and Casson models can be used to model flow curves with yield stress [94]. Barrios-Rodríguez et al. [28] observed that the Herschel–Bulkley and Casson models adequately described the flow behaviour of rice protein ink before and after 3D printing. The Herschel–Bulkley model was used to characterise the rheological properties of ink based on mashed potatoes [33] and milk [55].

Computational fluid dynamics is a modelling technique which has been used to relate the formulation, design process, and successful 3D printing of different foods [97], e.g., lemon juice gels [97] and gels with cereals [98].

### 3.2. Print Fidelity and Stability of Products

A final product’s structure is contingent on the quality of the food materials. Materials with a lower viscosity (10–500 cP) facilitate accelerated printing and yield smoother layers, while those with a higher viscosity (1000–5000 cP) are more conducive to the creation of intricate patterns [5]. The stability of an object’s shape during the printing process is contingent upon the stress level at which the material deviates from linear behaviour in an oscillatory stress sweep test. A pivotal factor is the yield strength, which dictates the capacity to extrude within the machine’s operational limits and the fidelity of the printed shape. To ensure structural stability, inks must adopt the characteristics of solids after extrusion, thereby facilitating the creation of self-supporting structures, minimising the risk of buckling or sagging and ensuring precise design reproduction [99].

Dick et al. [100] demonstrated that samples characterised by larger phase angles at higher frequencies exhibited a heightened propensity for viscous behaviour. This, in turn, resulted in a reduction in shape stability and printing accuracy. The concept of stability was articulated by assessing the modulus of elasticity (G′) and loss (G′), where extrudable samples exhibited G′ values ranging from 357 to 6487 Pa. In contrast, the control sample (bovine paste, 33 Pa) and bovine paste with k-carrageenan (19.957 Pa) proved to be non-printable. Samples demonstrating more significant dimensional deviation during the 3D printing process exhibited increasing phase angles, reaching values of 21.3 ± 0.8° and 23 ± 1°, indicative of diminished shape stability over time. Conversely, samples exhibiting minimal or insignificant deviations demonstrated a consistent or decreasing trend in the phase angles, ranging from 13.2 ± 0.2° to 17.4 ± 0.7°.

Studies were conducted on printed objects created using soy protein-based inks. Control samples showed deformation over time, leading to collapse (after 2 min), resulting in low shape fidelity and poor resolution for various shapes, including stars, hearts, and cylindrical objects. The higher flow rate exhibited by the control ink indicated less pronounced shear behaviour, making the embossing process more tedious [4]. Gup et al. [101] found that a microwave-treated sample with 1% calcium chloride exhibited the highest print fidelity (75.63 ± 0.60%), significantly higher than that of an only-microwave-treated sample. The printability of buckwheat starch gels with high-methoxy pectin was assessed by printing a 20 × 20 × 10 mm cube with four square holes. The control sample could not form a cube due to its low plasticity and high viscosity, which limited its printability.

## 4. Printing Parameters and Methods

A 3D model is created by bonding or depositing material layer by layer. This is achieved through a range of techniques, including selective laser sintering (SLS), fused deposition modelling (FDM), stereolithography (SLA), binder jet printing, and semi-solid extrusion [102]. In particular, extrusion is the most effective method of printing soft products such as pastry, meat purée, potato purée, and hot chocolate extrusion [103].

The food printing process is based on two groups of parameters: extrusion (temperature, shear force, pressure, screw speed, extrusion speed) and printing (table speed, layer thickness). The layers must be strong enough to avoid deformation. The film thickness depends on the table speed, extrusion speed, and die diameter; smaller dies allow thinner layers with better quality. Too fast a table speed can interrupt the flow of material, while too slow a table speed will cause material to build up and reduce the print quality [104].

One strategy for reducing costs in the printing process is to improve production efficiency. One standard method for increasing efficiency is to increase the printing speed and use larger nozzles or more powerful lasers. However, this methodology often results in decreased precision and resolution in printed objects, harming the quality of final 3D-printed food products. Therefore, balancing sufficient precision and using larger nozzles and faster printing is essential. An alternative method for increasing efficiency is to use printers with multiple nozzles, which allow for the simultaneous production of many objects [103].

Yang et al. [25] demonstrated that an elevated extrusion speed (28 mm^3^/s) resulted in the formation of wavy lines with augmented diameters, thereby leading to overlapping layers and substantial deviations when printing tall products. Conversely, a low extrusion speed (20 mm^3^/s) resulted in broken lines, a collapsed structure, and significant discrepancies between the design and the product. However, Wang et al. [29] conducted a study to ascertain the viability of 3D printing a surimi. In this investigation, a range of factors were examined, including the nozzle height (set at 5, 10, 15 or 20 mm), nozzle diameter (0.8, 1.5 mm, or 2.0 mm), extrusion speed (from 0.002 to 0.005 cm^3^/s), and nozzle traverse speed (20, 24, 28, and 32 mm/s). The optimal 3D printing parameters were determined as a high resolution, fitting to the target geometry, few point defects, and a lack of compression deformation. These parameters were achieved by using a nozzle diameter of 2.0 mm and a height of 5.0 mm, a nozzle movement speed of 28 mm/s, and an extrusion speed of 0.003 cm^3^/s. Using a reduced nozzle diameter enabled the researchers to obtain a higher print resolution. However, the printing time considerably increased with this method. It is necessary to identify an optimal balance between efficiency and precision in the printing process [103].

Printing temperature has been demonstrated to significantly impact key aspects of the sensory experience, including texture, taste, colour, and overall quality. However, limitations related to the print size and speed constrain the application of this technology in mass food production. To address these challenges, it is necessary to optimise printing methods and improve material flow dynamics and deposition procedures to speed up printing without compromising product quality and structural integrity [5]. Martínez-Monzó et al. [24] conducted a study to analyse the effect of temperature (10 °C, 20 °C, and 30 °C) on the 3D printing process of potato purée with milk. The results indicated that the optimal temperature for the printing process was 30 °C. The potato-based ink demonstrated optimal stability at this temperature; the effect of temperature on the product was related to its composition. García-Segovia et al. [105] reported the effect of temperature (25 and 50 °C) on the printability of gels based on syrup, xanthan gum, and konjac. The results indicated that gels printed at 50 °C exhibited reduced flexibility and higher fluidity [105]. Hamilton et al. [106] investigated the effect of temperature and pressure variation on the 3D printing of a formulation with breakfast paste and toasted bread. Applying a pressure of 172 kPa resulted in the extrusion of both materials at 25 °C. This pressure was insufficient at a temperature of 45 °C and caused the material to flow too quickly, leading to fluid leakage. The decrease in pressure to 103 kPa enabled the improvement of the process. Furthermore, an increase in temperature to 65 °C resulted in premature extrusion, even at very low pressures (<34 kPa) [106].

### 4.1. Jetting-Based Method

**Figure 2:**
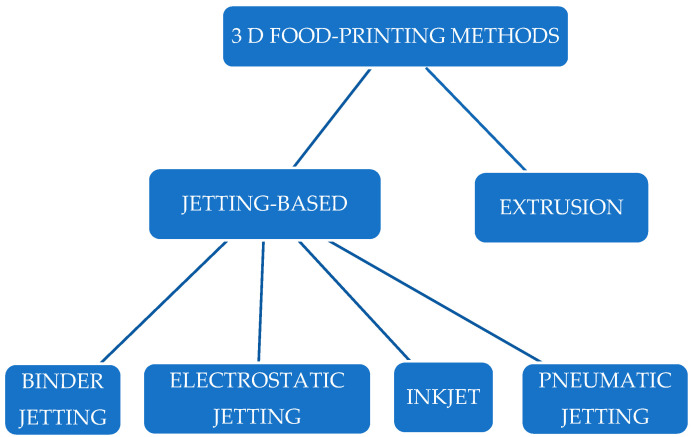
Classification of jetting-based techniques within the field of three-dimensional printing.

Three-dimensional jet printing involves the deposition of liquid materials layer by layer, each consisting of individual droplets. The droplets are then applied to a substrate, a previous layer of the same material, or a layer of powder. Combining several of these layers creates a 3D object. These techniques are distinguished by higher resolution and better control over the amount of material applied. Similarly to the extrusion method, these techniques are characterised by relatively small application units, which are drops rather than lines. These techniques include piezoelectric inkjet printing, bonded printing, electrostatic printing, hot foil printing, and pneumatic printing. A classification of 3D jet printing techniques can be seen in Figure 2 [14].

### 4.2. Extrusion Method

Three-dimensional extrusion food printing is widely popular due to its simplicity and versatility. It can be used with various food materials, including cake, chocolate, meat substitutes, and vegetable pastes [5]. In extrusion printing, solids, semi-solids, and liquids are forced through a die opening, producing a product with a constant cross-section. The process involves decomposing raw materials and forming the final product, facilitated by continuous operation and high efficiency. These printers can be classified according to differences in temperature control: hot-melt extrusion (HME), hydrogel-forming extrusion (HFE), and room-temperature extrusion (RTE) [104].

A significant challenge is to regulate the material’s flow properties for precise deposition. The study of rheological properties is a valuable tool for optimising printing processes, as it allows for understanding the viscoelastic behaviour of the materials in question. The nozzle’s dimensions, in addition to its configuration, affect the resolution and speed of the printing process. Optimal nozzle design with the right temperature prevents obstructions and maintains the material’s structural integrity. Understanding the interactions between layers and additives, such as gelling agents and stabilisers, facilitates the refinement of the printing process. In addition, post-print heat treatment and dehydration are essential to achieve the desired texture and flavour [5].

## 5. Innovative Use of Food Inks

An innovative product that uses 3D printing is plant-based meat analogues that replicate meat’s structure, taste, and appearance. This is an important factor in sustainably feeding a growing population while mitigating the impact of animal farming on the environment [107]. Demircan et al. [108] developed meat analogues for 3D printing with plant-based protein sources enriched with three types of mushrooms: reishi, saffron milk-cap, and oyster mushrooms. Incorporating mushrooms resulted in enhanced juiciness, reduced hardness and stiffness, augmented nutritional value, and the release of umami amino acids. Adding mushrooms made the meat analogues more appealing and nutritious for vegan consumers.

The demand for multifunctional structures has led to interest in developing responsive solutions. Four-dimensional printing enables structures that differ from 3D ones in that they can “morph” or change over time. Such structures, such as pinecones and carnivorous plants, are observed in nature. Such structures respond to a wide range of stimuli. Recent advances have enabled the development of simple, passively responsive systems. To achieve responsive systems, two basic requirements must be met: the material used for printing must respond to external stimuli, and there must be a process that allows for the controlled distribution of this material [109]. Four-dimensional printing (4D printing) represents a significant advancement in food printing, extending the capabilities of three-dimensional food printing to encompass the “space-time axis”. This innovative technology enables food to change shape, functionality, and properties in response to external stimuli. The application of 4D printing in food products empowers the creation of dynamic structures that are aesthetically pleasing. It also offers a multi-faceted sensory experience while reducing capital requirements, minimising storage space, increasing operational efficiency, and reducing lead time [110]. Four-dimensional printing offers a more immersive sensory experience compared to 3D printing. Flowering flowers are such an example. The transformation of a material from a closed to a blooming state enhances the appeal of the food, attracting attention, particularly for children who are picky about food. A notable advantage of 4D printing over 3D printing is its ability to release a product’s flavour, nutritional value, or colour only when consumed rather than during storage [110,111].

The introduction of 4D printing technology enables the incorporation of an element of surprise. It significantly enhances the variety of food products, which is particularly beneficial for consumers with specific dietary requirements or preferences, such as children who are fussy about their food. The printed product’s taste, colour, or nutritional value is released only during consumption, not storage [111].

The interaction between food inks and external stimuli represents a challenge for 4D printing. The structure and properties of stimuli-responsive components must be considered when designing printed food to ensure that it responds to external stimuli over time. Successful implementation depends on adjusting factors like printing technology, digital models, food inks, and post-production processes. Four-dimensional food printing is based on three-dimensional printing. When printing food inks, paying attention to the filling speed, extrusion speed, nozzle diameter, and layer height is essential. These affect the quality and response to stimuli [110]. The external stimuli employed in 4D printing can be categorised into three principal groups: pH potential, temperature, and light source. Colour is one of the most significant factors influencing consumer choice, particularly in the context of food products. It has been demonstrated that using natural pigments and pH adjustments can affect the signals transmitted by receptor sources and stimuli in 4D printing [112]. An exemplar of this phenomenon is curcumin, which exhibits a red colouration at an alkaline pH and a yellow hue at an acidic or neutral pH [113].

Kang et al. [114] demonstrated the broader development of 4D-printed products utilising insect proteins in the food industry. The investigation involved the utilisation of cricket powder and a purée of purple potatoes as the primary ingredients for 4D printing, with a colour change in the material in response to pH alterations (4D). Ghazal et al. [115] investigated the 4D changes in the colours and flavours of 3D-printed foods in response to external or internal stimuli, such as pH. Materials used in the study included red cabbage juice, vanillin powder, potato starch, and fruit juices. The colour of the products changed from blue to red, purple, blue, blue–green, and green–yellow depending on the pH (2–10). There were also differences in aroma and flavour.

Concerning the alteration in the colour of the product, Chen et al. [116] investigated the colour change in a 4D-printed lotus root powder gel under microwave stimulation, with curcumin serving as the stimulus-response material. Initially, the sample exhibited a yellow hue, which transitioned to red following microwave stimulation. With regard to alteration in product taste, Phuhongsung et al. [117] developed a blend of soy protein isolate (SPI), k-carrageenin (CAR), and vanilla flavouring for 4D printing, utilising microwave heating as an external stimulus. The study found that the taste of the printed products was affected by different levels of microwave irradiation power. A high pH of 10 enhanced the saltiness of a 3D-printed soy protein isolate blend with pumpkin and beets. The study’s significant point was the effects of varying pH levels on this composite mixture’s colour, texture, and taste. Stimulation at a pH of 8 and 10 significantly influenced the flavour profile, while a pH of 4 and 10 led to alterations in taste, resulting in astringency and saltiness, respectively [118].

Concerning alteration in texture, Ghazal et al. [115] observed that the agglomeration of protein molecules at elevated pH levels enhanced the elasticity and cohesiveness of a gel. In contrast, the application of solutions at different pH values (2–10) to a mixture of 3D-printed red cabbage juice, vanillin powder, and potato starch had a detrimental effect on the textural properties of the mixture, reducing its hardness, elasticity, and gumminess in comparison to the control samples. Samples at a pH of 3 exhibited the highest texture and rheological values, which may be attributed to the leaching of starch grains. The study showed no significant differences in textural parameters among samples at varying pH levels. The researchers modified the final shape and duration of the cooking time, offering customers an innovative sensory experience [119]. Shi et al. [120] employed microwave heating as an external stimulus to study the oligo-gel and purée of purple potatoes. The study reported a shape change in the purée and the oligo-gel caused by the microwave treatment. The linear relationship between the microwave power, time, and bending angle was observed for the purple potato purée.

So far, 4D printing research has mainly focused on modifying a single property of a product using a single stimulus-responsive material. It is inevitable, however, that it will be necessary to develop combinations of multiple types of such materials to direct changes in different properties in the future [115].

The disadvantages of 4D printing include the limitations of printing techniques, the materials used for printing, and the techniques applied for designing structures. Conversely, 4D printing offers a future-proof solution; however, further research is required to identify more intelligent materials and innovative, efficient printing technologies [121].

Ensuring the stability of a material and the sensory quality of the product and that these characteristics are not undesirably changed by post-production is a major challenge for 4D printing technology. The solution may lie in developing more suitable post-production methods to improve efficiency and even achieve colour change and deformation in printed food [122]. Most current research on 4D printing is focused on using soy protein starch isolate and hydrogels, emphasising exploiting the material’s inherent properties, internal structural design, and spatial configuration. Therefore, 4D food printing is still in the developmental and research phase. Thus, this technology has excellent potential for further improvement [111]. Consumer acceptance should be a key consideration for further study [122]. To date, the effects of microwave heating and pH changes have been the primary focus of study; however, future research may encompass novel stimuli such as light or alterations in ion concentration. The development of new stimuli-responsive materials, such as diacetyl or vanillin, and their utilisation in 4D food printing will facilitate the achievement of simultaneous changes in multiple food properties, representing a breakthrough in this technology [113].

## 6. Conclusions

Using 3D printing technology in the food industry is gaining popularity, offering numerous benefits, including the ability to meet the needs of a diverse group of consumers by producing custom shapes and providing products for people with swallowing disorders. However, developing suitable food inks with the right rheological and textural properties for 3D printing applications is still a significant challenge. The main goal of this study was to thoroughly analyse the composition of food inks for 3D printing. It was shown that adding appropriate thickening and gelling additives, such as hydrocolloids, improves the quality of inks. In addition, adjustments to parameters such as temperature, printing speed, and nozzle size are necessary to ensure the stability and durability of 3D-printed products. Three-dimensional printing remains a significant field of research and development in the food industry. A significant consideration for the future is the application of innovative 3D printing solutions, such as the production of plant-based meat analogues. These inks will substantially impact the environment and the sustainable feeding of a growing population. Another promising development is 4D printing, an extension of 3D printing. Materials can transform in response to diverse stimuli, altering their shape, functionality, and properties. This renders them all the more appealing from the standpoint of sensory experience. Designing inks for 4D printing is difficult and requires knowledge of the behaviour of individual components in ink products after a given stimulus. Nevertheless, further research is required in this area.

## Figures and Tables

**Table 1:** Three-dimensional printing ink compositions and their parameters.

Food Materials	Printing Method	Nozzle Diameter	Nozzle Height	Process Temperature	Printing Speed	Rheological Characteristic	Ref.
Chilled chicken breast (mince) and edible salt	Extrusion	0.8, 1.0, 1.2, 1.5, and 2.0 mm	Consistent with nozzle diameter	25, 35, and 45 °C	25 mm/s	Non-Newtonian and pseudoplastic	[20]
Cheese	Extrusion	1.5 mm	75 mm	180 °C	A computer controlled the motion and positioning of the system	Non-Newtonian and pseudoplastic	[21]
Cricket powder (*Acheta Domesticus*) and xanthan gum	Direct ink writing based on pneumatic extrusion	0.9 mm	X	25 ± 1 °C	A computer controlled the motion and positioning of the system	Non-Newtonian and pseudoplastic	[22]
Dark chocolate and two types of additives: magnesium stearate powder and plant sterol powder	Extrusion	0.78 mm	X	32 °C	70 mm/s	Non-Newtonian and pseudoplastic	[23]
Dehydrated potato purée and whole milk	Extrusion	2 mm	2 mm	10, 20, and 30 °C	2 mm/s	Non-Newtonian and pseudoplastic	[24]
Dough (caster sugar, butter, low-gluten flour, egg, and water)	Extrusion	0.8, 1.5, and 2 mm	0.84 mm	24.8 °C	25 mm/s	Non-Newtonian and pseudoplastic	[25]
Milk powder	Direct ink writing based on pneumatic extrusion	0.5–1.3 mm	X	25 ± 1 °C	A computer controlled the motion and positioning of the system	Non-Newtonian and pseudoplastic	[26]
Paste: starch, milk powder, cellulose nanofibers, rye bran, oat protein concentrate, and bean protein concentrate.	Extrusion	0.41 mm	X	25 ± 1 °C	2 mm/s	Non-Newtonian and pseudoplastic	[19]
Puréed vegetables (carrots, pak choi, and garden peas) and hydrocolloids (kappa carrageenan, locust bean flour, and xanthan gum)	Extrusion	0.84 mm	0.5 mm	25 ± 1 °C	25 mm/s	Non-Newtonian and pseudoplastic	[27]
Rice protein isolate, water, and xanthan gum	Extrusion	1,2 mm	X	25.5 ± 0.5 °C	50 mm/s	Non-Newtonian and pseudoplastic	[28]
Surimi fish gel	Extrusion	0.8, 1.5, and 2.0 mm	5, 10, 15, and 20 mm	25 °C	Different extrusion speeds for various movement speeds	Non-Newtonian and pseudoplastic or thixotropic	[29]
Textured soy protein, onion, breadcrumb powder, xanthan gum, gluten powder, salt, spices, and sunflower oil	Extrusion	4.0 mm	4.0 mm	25 ± 1 °C	5–10 mL/s	Non-Newtonian and pseudoplastic	[30]
Whey protein powder, peanut protein powder, casein, hydrolysed wheat protein, and pea protein powder	Extrusion	0.8; 1.0; 1.2; and 1.5 mm	The printing layer height was the nozzle diameter (0.8, 1.0, 1.2, and 1.5 mm)	25 ± 1 °C	2 mm/s	Non-Newtonian and pseudoplastic	[31]
Egg-white protein, tea polyphenols, polyphenols, and phosphate-buffered saline	Extrusion	1.2 mm	X	25 °C	15 mm/s	Non-Newtonian and pseudoplastic	[32]
Ground purée and 15% trehalose	Extrusion	2.0 mm	3.0 mm	25 °C	The nozzle speed and extrusion rate were adjusted to achieve the desired results	Non-Newtonian and pseudoplastic	[33]
Ground purée, sodium alginate, citric acid, and sodium bicarbonate	Extrusion	1.2 mm	1.2 mm	25 °C	15 mm/s	Non-Newtonian and thixotropic	[34]
Lemon juice and ground starch	Extrusion	0.5, 1.0, 1.5, and 2.0 mm	X	25 ± 1 °C	24 mm/s	Non-Newtonian and pseudoplastic	[35]
Low-methoxylated pectin + CaCl_2_, bovine serum albumin, and sugar syrup	Extrusion	0.84 mm	0.84 mm	25 ± 1 °C	10 mm/s	Non-Newtonian and pseudoplastic	[36]

X—no data.

## Data Availability

No new data were created or analyzed in this study. Data sharing is not applicable to this article.
